# Role of satellite glial cells in gastrointestinal pain

**DOI:** 10.3389/fncel.2015.00412

**Published:** 2015-10-13

**Authors:** Menachem Hanani

**Affiliations:** Laboratory of Experimental Surgery, Hadassah-Hebrew University Medical Center, Mount ScopusJerusalem, Israel

**Keywords:** dorsal root ganglia, nodose ganglia, chronic pain, colonic inflammation, satellite glial cells, gap junctions, purinergic receptors

## Abstract

Gastrointestinal (GI) pain is a common clinical problem, for which effective therapy is quite limited. Sensations from the GI tract, including pain, are mediated largely by neurons in the dorsal root ganglia (DRG), and to a smaller extent by vagal afferents emerging from neurons in the nodose/jugular ganglia. Neurons in rodent DRG become hyperexcitable in models of GI pain (e.g., gastric or colonic inflammation), and can serve as a source for chronic pain. Glial cells are another element in the pain signaling pathways, and there is evidence that spinal glial cells (microglia and astrocytes) undergo activation (gliosis) in various pain models and contribute to pain. Recently it was found that satellite glial cells (SGCs), the main type of glial cells in sensory ganglia, might also contribute to chronic pain in rodent models. Most of that work focused on somatic pain, but in several studies GI pain was also investigated, and these are discussed in the present review. We have shown that colonic inflammation induced by dinitrobenzene sulfonic acid (DNBS) in mice leads to the activation of SGCs in DRG and increases gap junction-mediated coupling among these cells. This coupling appears to contribute to the hyperexcitability of DRG neurons that innervate the colon. Blocking gap junctions (GJ) *in vitro* reduced neuronal hyperexcitability induced by inflammation, suggesting that glial GJ participate in SGC-neuron interactions. Moreover, blocking GJ by carbenoxolone and other agents reduces pain behavior. Similar changes in SGCs were also found in the mouse nodose ganglia (NG), which provide sensory innervation to most of the GI tract. Following systemic inflammation, SGCs in these ganglia were activated, and displayed augmented coupling and greater sensitivity to the pain mediator ATP. The contribution of these changes to visceral pain remains to be determined. These results indicate that although visceral pain is unique, it shares basic mechanisms with somatic pain, suggesting that therapeutic approaches to both pain types may be similar. Future research in this field should include additional types of GI injury and also other types of visceral pain.

## Introduction

The term “visceral pain” covers a wide range of pain types associated with internal organs located in the thorax and abdominal cavity. Visceral pain is a major clinical problem and is “the most frequent form of pain produced by disease and one of the main reasons for patients to seek medical attention” (Cervero and Laird, 2004). Among the types of visceral pain, gastrointestinal (GI) pain is probably the most common, and is associated with a large number of GI disorders like gastritis, celiac disease, pancreatitis, constipation, and biliary tract inflammation. A major group of GI problems comprises syndromes known as “functional GI disorders”. These syndromes include irritable bowel disease (IBS) and functional dyspepsia, and are characterized by pain, but have no identifiable structural or biochemical abnormalities (Chang et al., 2010). Patients with inflammatory bowel disease (colitis, Crohn’s) frequently complain of abdominal pain, which may persist after the resolution of inflammation, indicating that long term changes in the nervous system contribute to this post-inflammatory pain (Bielefeldt et al., 2009; Beyak, 2010). Stimuli that evoke GI pain can be mechanical, like distension of hollow organs (stomach, intestine), and traction of the mesentery; or chemical, like acid. Ischemia and various inflammatory mediators can also cause visceral pain, or to lower the pain threshold to mechanical stimuli. For reviews on this topic see Cervero and Laird (2004); Bielefeldt and Gebhart (2013). The mechanisms underlying many types of GI pain are far from being understood and pain management options continue to remain limited (Bielefeldt et al., 2009).

GI pain is typically diffuse and non-localized and not necessarily felt at the source of stimulus or injury (Cervero and Laird, 2004; Bielefeldt and Gebhart, 2013). A well known example for referred pain is angina pectoris, which is caused by cardiac hypoxia, and may cause pain in the left arm. A possible explanation for these and other types of referred pain is that the nerve supply to these internal organs develops during the embryonic period from the respective skin regions. Still, this explanation is far from being mechanistic.

## Sensory Ganglia and Visceral Pain

The initial and often driving force for somatic pain is peripheral injury or inflammation (Devor, 2013), and therefore understanding peripheral mechanisms should be the focus of pain research (in distinction from the current emphasis on central mechanisms). A reasonable working hypothesis is that the same principle also holds for visceral pain, and clearly sensory ganglia, which are the first station in the pain pathway, deserve to be a major subject for studying visceral pain, and are a preferred target for its treatment. Thus, the account below is devoted to discussing the involvement in GI pain of neurons and glial cells in sensory ganglia.

**Figure 1:**
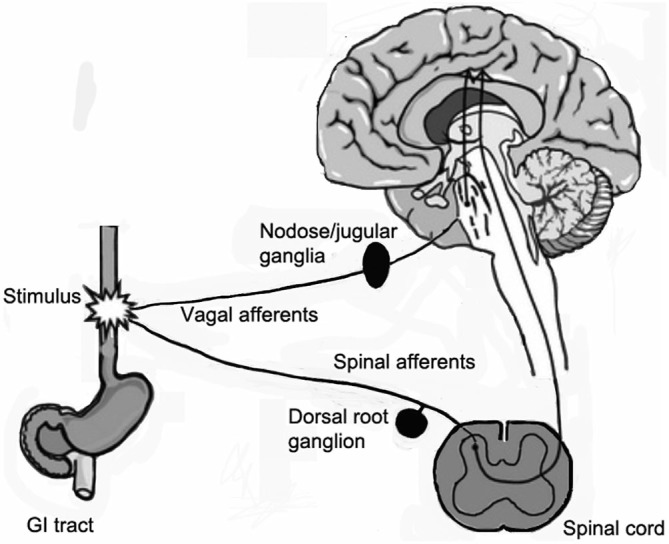
**Diagram showing the sensory innervation of the gastrointestinal (GI) tract.** The GI tract sends afferents to spinal cord by spinal nerves via dorsal root ganglia (DRG), and vagal afferents, which originate from neurons in the nodose/jugular ganglia and reach the brain stem.

Sensory ganglia contain the cell bodies of primary afferent neurons, which have a bifurcating axon, with one branch projecting to the periphery, and the other into the spinal cord (Figure 1). The main types of sensory ganglia are the dorsal root ganglia (DRG), which innervate most parts of the body, including internal organs; the trigeminal ganglia (TG), which innervate the head, face and teeth, and the nodose ganglia (NG), which innervate most visceral organs. Research in the last two decades has shown that despite their apparent simplicity, sensory ganglia are the site of neural information processing (Devor, 1999; Krames, 2015). Although devoid of synapses, sensory neurons possess numerous receptors for neurotransmitters and hormones and also release neurotransmitters, such as glutamate, ATP, substance P, and CGRP (Huang and Neher, 1996; Devor, 1999; Matsuka et al., 2001; Huang et al., 2013). Thus, the neurons can communicate among themselves and also with other cell types in the ganglia, mainly glial cells (Amir and Devor, 1996; Oh and Weinreich, 2002). Indeed, there is evidence for bidirectional interactions between neurons and glial cells in these ganglia (Suadicani et al., 2010; Huang et al., 2013), largely mediated by purinergic P2 receptors and gap junctions (GJ), but the significance and precise mechanisms of such communication is still obscure.

It is well established that under pathological conditions sensory neurons can generate spontaneous electrical activity, and thus play an active role in chronic pain (Cherkas et al., 2004; Devor, 2013; Hoeijmakers et al., 2015). It was reported that the sensitivity of sensory neurons to ATP, acting on purinergic P2X3 receptors (P2X3Rs; Zhou et al., 2001; Chen et al., 2005), and to substance P (Xu and Zhao, 2001) is increased in pain models, which may contribute to neuronal sensitization. There is evidence that also in rodent models of visceral pain DRG neurons undergo profound physiological and pharmacological changes (Moore et al., 2002; King et al., 2009; Beyak, 2010; Bielefeldt and Gebhart, 2013). Most notable are the changes in the expression and function of Na^+^ channels, resulting in augmented Na^+^ currents, which partly underlie the hyperexcitability of DRG neurons (King et al., 2009; Bielefeldt and Gebhart, 2013). Decreased K^+^ currents were also found to contribute to neuronal excitability in models of visceral pain (Dang et al., 2004; Beyak, 2010), leading to a greater excitability of sensory neurons and to pain.

As mentioned above, visceral organs can cross-sensitize by mechanisms believed to be due to convergence in spinal cord neurons (Bolser et al., 1991; Qin et al., 2004; Brumovsky and Gebhart, 2010), but the possibility of more distal contribution to this effect has received little attention. However, neuron-neuron cross talk within DRG does exist (Devor and Wall, 1990; Amir and Devor, 1996; Devor, 2013), suggesting that interactions at the DRG level may contribute to referred pain. Moreover, there is evidence for dichotomizing DRG neurons, with an axon that bifurcates and sends one branch to the colon, and the other to the bladder, which may account for referred pain between these two organs (Christianson et al., 2007). Another study showed that up to 40% of the axons of T3–T8 DRG neurons in rats dichotomize to both duodenum and pancreas (Li et al., 2013); for a review, see Brumovsky and Gebhart (2010). Still, direct functional evidence for the role of DRG in referred pain is lacking.

## Neuron-Glia Interactions in Pain

It has been found that glial cells (astrocytes and microglia) in the spinal cord undergo activation (reactive gliosis) following peripheral injury in animal models of chronic pain, suggesting the involvement of these cells in pain. This has led Watkins and her colleagues to state that the frequent failure of the available therapies for chronic pain is due to the emphasis on neurons as the target for analgesic drugs. They proposed that glial cells could be a better target for pain therapy (Watkins and Maier, 2002). Recent studies have provided firm support for this idea (Ji et al., 2013; Tsuda et al., 2013a,b). We suggested that not only central glial cells are involved in pain generation and maintenance, but also satellite glial cells (SGCs) in sensory ganglia play such a role (Hanani et al., 2002; Huang et al., 2010).

It has been stated that “changes in the excitability of primary nociecptive afferents are the single most important factor in the generation and maintenance of chronic neuropathic pain in humans” Scadding and Koltzenburg (2013). This conclusion holds also for central sensitization, because it is driven by peripheral changes. It is established that neuronal activity in DRG depends to a large degree on neuron-glia interactions (Hanani, 2012; Huang et al., 2013; Ji et al., 2013). Therefore learning on these interactions is essential for full understanding all types of pain, including visceral pain.

**Figure 2:**
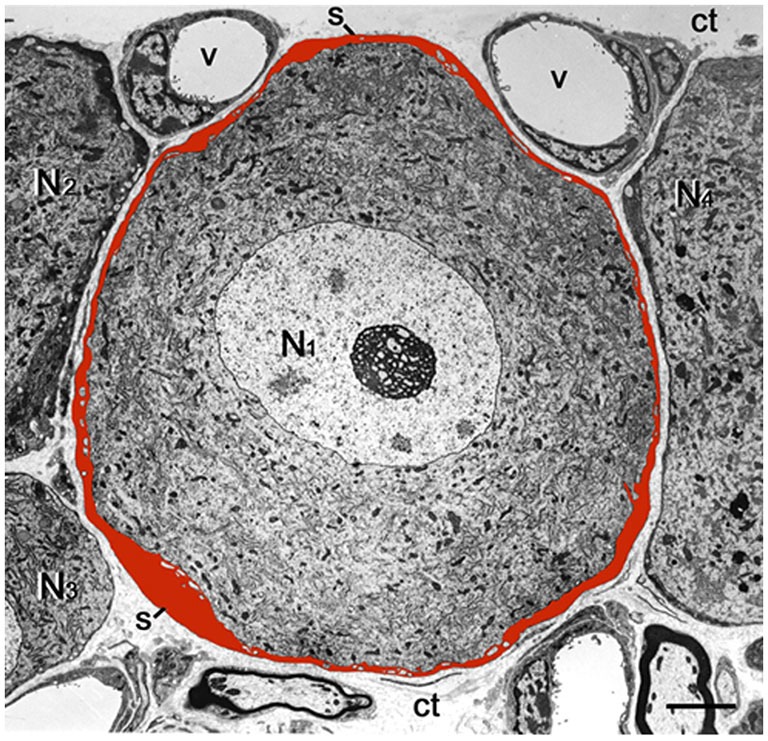
**Low magnification electron micrograph of mouse DRG showing the cell body of a sensory neuron (N1) enveloped by a satellite glial cell (SGC) sheath, which was painted red.** Note that the entire outer contour of the sheath is smooth and is completely separated from the sheaths encircling the adjacent nerve cell bodies (N2–N4) by the connective tissue space (ct). The small empty regions within the SGCs envelope are fine neuronal protrusions that increase the contact between the two cell types (see Pannese, 2002). s, SGS; v, blood vessel. Scale bar, 4 μm.

Neurons in sensory ganglia are surrounded completely by a tight envelope of SGCs, and it was proposed that the neurons and their surrounding SGCs form a distinct functional unit (Hanani, 2005), see Figure 2. The gap between the neurons and SGCs is only 20 nm, and therefore the neuronal extracellular space is very small, which enables a tight control of the neuronal environment by the SGCs (and neurons). The SGCs sheath around a given neuron is complete, but is permeable even to large molecules, such as proteins (for review, see Hanani, 2005). Still, as SGCs are endowed with transporters for various neuroactive molecules (Hanani, 2005; Jasmin et al., 2010), the glial envelope can function at least as a partial barrier between the circulation and the neurons. The DRG vasculature is fenestrated, and therefore there is no blood-ganglion barrier (Hanani, 2005). A subpopulation (1.6–9.4%, depending on species and age) of DRG neurons are not surrounded by SGC sheath singly, but two or more of neurons have a common glial cover, forming an arrangement termed “cluster”, (Pannese et al., 1991, 1993). For reviews on SGCs (see Pannese, 1981, 2010; Hanani, 2005; Takeda et al., 2009; Jasmin et al., 2010; Hanani, 2012; Hanani and Spray, 2013; Huang et al., 2013; Ji et al., 2013). There is considerable information on how SGCs are altered in models of somatic and orofacial pain models (see references above), but much less is known about these cells in models of visceral pain. Below is an account of the current knowledge on this topic.

## SGCs and Pain in Partial Colonic Obstruction

Huang and Hanani (2005) reported that in a mouse model of partial colonic obstruction, the threshold for withdrawal response to mechanical stimulation of the abdominal skin was markedly reduced compared with controls. It has been established that this response is a reliable indication for GI pain (Laird et al., 2001). We found by retrograde labeling that the distal colon is innervated predominantly by S1 and L1 DRG neurons, and these ganglia were selected for the *in vitro* study. Intracellular electrical recordings from neurons in ganglia obtained from mice after partial obstruction showed an augmented frequency of spontaneous subthershold oscillations, which are an important contributing factor in the generation of neuronal hyperexcitability in pain models (Amir et al., 1999; Devor, 2013). DRG neurons from obstructed mice also displayed a greater degree of spontaneous action potentials, which are likely to be due to the subthrehold oscillations. Intracellular injection of the dye Lucifer yellow (which crosses GJ) into individual SGCs showed that SGCs from DRG of obstructed mice displayed augmented coupling via GJ (Huang and Hanani, 2005). Most prominent was the increased coupling between SGC envelopes around different neurons, which has the potential to strengthen the communication between the neuron-SGC units. The augmented coupling was observed in cells injected randomly throughout the ganglia, but when dye was injected to SGCs near neurons projecting to the distal colon, coupling between glial envelopes was over 3-fold greater compared with random injection. As only 1–2% of neurons in the ganglia project to the distal colon, the effect of obstruction must have spread from directly-affected neurons to non-affected ones, but still SGCs near neurons directly affected displayed a greater degree of coupling. It may be concluded that the affected neurons release factors that can diffuse and influence neighboring cells. Such spread of glial activation in sensory ganglia following peripheral damage is well documented (e.g., Stephenson and Byers, 1995; Thalakoti et al., 2007), but the released factors are not yet known.

The nature of the injury in the obstruction model is not well-defined, but it is probably associated with local inflammation because the density of neutrophils was elevated within the colonic muscles. Also, the colonic wall was thickened (both muscle and mucosa) as found in models of intestinal inflammation in rats (Eskandari et al., 1997; Moreels et al., 2001). These findings suggested that inflammatory processes within the intestinal wall triggered the changes in the DRG that contributed to chronic pain. The observation of the increased SGC coupling led to the suggestion that this and other changes in these cells may contribute to augmented neuronal excitability and to GI pain (Huang and Hanani, 2005).

## SGCs in Colonic Inflammation

**Figure 3:**
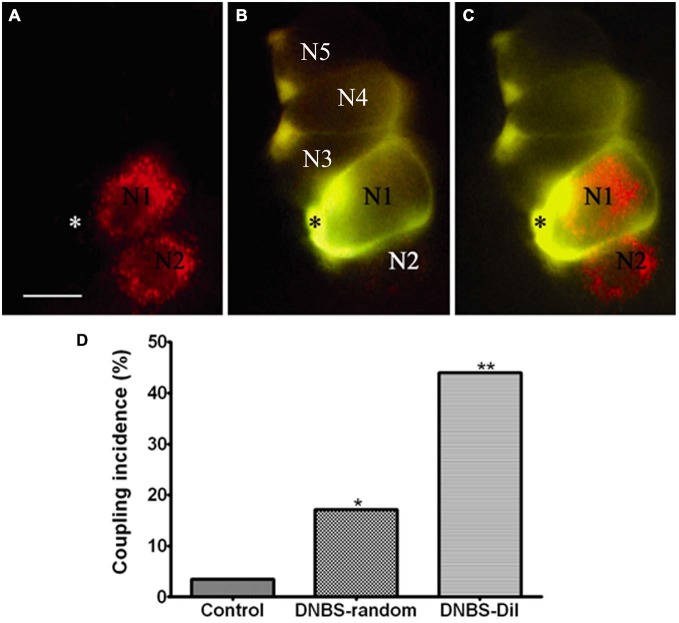
**Inflammation-induced changes in dye coupling between SGCs in the vicinity of neurons that innervate the colon. (A)** A micrograph showing two DiI-labeled neurons (marked N1, N2), which innervate the colon. **(B)** An SGC (asterisk) near N1 was injected with LY. **(C)** Merged image obtained from **(A)** and **(B)** showing the relationship between DiI-labeled neurons and LY-injected SGC. Note that the LY-injected SGC is coupled to other SGCs that ensheathe unlabeled (presumably non-colonic) neurons. The images are of living cells during the injection experiment. Scale bar, 25 μm. **(D)** The histogram shows that SGCs coupling increased after the induction of inflammation. The effect was much greater when the dye-injected SGCs were in the vicinity of the DiI-labeled neurons compared with SGCs that were injected randomly throughout the ganglion. *p < 0.05, **p < 0.01. Modified from Huang et al. (2010).

**Figure 4:**
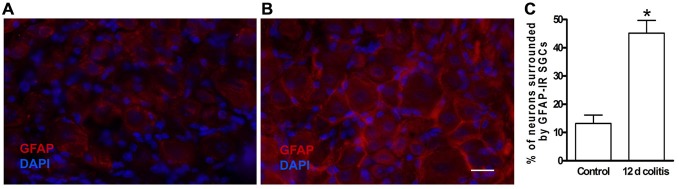
**Glial fibrillary acidic protein (GFAP) expression is increased in SGCs in dorsal root ganglion DRG of dinitrobenzene sulfonic acid (DNBS)-treated mice. (A)** Control. **(B)** The labeling of SGCs for GFAP 10–12 days following colonic inflammation was greatly increased. **(C)** Quantitation of GFAP expression in SGCs in DRG from controls and from DNBS treated animals (12 d colitis). The upregulation of GFAP indicates the activation of SGCs. *p < 0.05.

The account above suggested a possible involvement of SGCs in the events connecting the changes in the colon to neuronal hyperexcitability in the obstruction model. We tested this idea in a study where colonic inflammation was induced by instilling dinitrobenzene sulfonic acid (DNBS) into the lumen of the distal colon of mice (Huang et al., 2010). The ganglia (S1 DRG, which innervate the distal colon) were examined *in vitro* 10–12 days after DNBS application. First we established that the DNBS-treated mice displayed tactile hypersensitivity in response to stimulation of the abdominal skin. As found in models of somatic (Hanani et al., 2002; Dublin and Hanani, 2007) and orofacial (Cherkas et al., 2004) pain and also in the obstruction model, dye coupling of SGCs around different neurons was greatly augmented (by 6.6-fold, Huang et al., 2010), see Figure 3. GFAP expression in SGCs was upregulated by nearly 4-fold in this model (Hanani and Belzer, 2014), see Figure 4. To learn about the nature of the SGC coupling, we incubated the ganglia with the gap junction blocker carbenoxolone, which blocked the dye coupling, confirming that it was mediated by GJ. It is known that carbenoxolone has various pharmacological actions besides blocking GJ, and for further confirmation we used (separately) two additional gap junction blockers, palmitoleic acid and meclofenamic acid, which are chemically distinct from carbenoxolone and from each other. Meclofenamic acid was found to have some inhibitory actions on neurons (Peretz et al., 2007), whereas there is no report on the direct effect of palmitoleic acid in neurons. These blockers again inhibited the dye coupling. The fact that all these three established gap junction blockers have the same actions of neuronal excitability is highly suggestive that their main effect was to reduce neuronal excitability through the blockade of GJ. It should be emphasized that the experiments were done in intact, freshly isolated ganglia, where the SGC-neuron unit is not disrupted, as may occur in isolated or cultured cells (see Belzer et al., 2010). Using intracellular electrical recordings from S1 DRG neurons, we verified that 10–12 d after the induction of colonic inflammation the neurons were hyperexcitable, as evidenced by a lower depolarizing current that was needed to evoke an action potential and by the increased spontaneous activity (both subthreshold oscillations and spontaneous action potentials).

**Figure 5:**
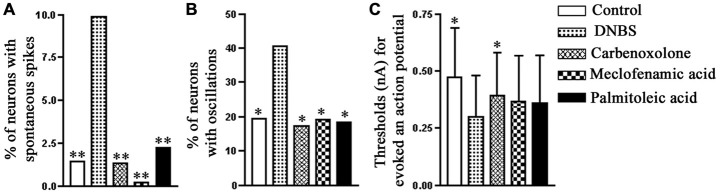
**The effect of gap junction blockers on the electrophysiological properties of neurons in mouse S1 DRG after colonic inflammation.** Ten to twelve days after the induction of inflammation, the proportion of the neurons with spontaneous action potentials **(A)** and spontaneous potential oscillations **(B)** increased, and also there was a decrease in the threshold for firing an action potential **(C)**. All the three gap junction blockers, carbenoxolone (50 μM), meclofenamic acid (100 μM) and palmitoleic acid (30 μM), applied separately, effectively reversed the neuronal hyperexcitability induced by the colonic inflammation. The comparisons are between data obtained for the DNBS-treated mice and data obtained for the other experimental groups. *p < 0.05, **p < 0.01, Fisher’s exact test. Modified from Huang et al. (2010).

We then asked whether the augmented SGC coupling and the neuronal hyperexcitability were related causally, and to answer this question we repeated the electrical recordings from neurons in the presence of gap junction blockers. The results showed that each of the three gap junction blockers mentioned above was able to normalize the neuronal behavior (Figure 5). That is, blocking SGC GJ restored neuronal excitability to control level. As the experiment was done on isolated DRG, the blockers must have acted directly on DRG cells (most likely on SGCs). It should be added that DNBS also increased neuron-neuron coupling; this was observed in only 12.1% of the neurons (compared with 0.7% in controls), but could have contributed to the overall effect. Neuron-SGC coupling has been reported for rat TG after application of capsaicin to the neuronal terminals in the skin (Thalakoti et al., 2007). We observed neuron-SGC coupling in only 1.6% of the cases in treated animals, but this is probably an underestimate because of the difficulty in detecting such coupling. Finally we asked whether blocking GJ in behaving mice could reduce the abdominal tactile hypersensitivity (which reflects colonic pain). We injected (i.p.) each of the three gap junction blockers (separately) 1 h before testing pain behavior, and found that this elevated the threshold in DNBS-treated mice, consistent with an analgesic effect of the blockers. As the gap junction blockers were injected systemically, the site of their action could not be determined, but there is evidence that carbenoxolone and meclofenamic acid are hydrophilic, and are unlikely to cross the blood brain barrier, which was directly confirmed for carbenoxolone (Leshchenko et al., 2006). To address this point we showed in a subsequent study that in DRG that were removed from animals injected i.p. with carbenoxolone, the GJ (induced in a pain model) were still blocked (Warwick and Hanani, 2013). This was interpreted as an evidence that carbenoxolone injected i.p. binds to SGCs and that the binding and the gap junction blockade persisted even after the removal of the ganglia from the body.

Another study on the effect of colonic inflammation was carried out by Song et al. (2014), who induced colonic inflammation in rats with 2, 4, 6, trinitrobenzene sulfonic acid (TNBS). They reported that 4 d after TNBS administration, GFAP immunostaining in SGCs was greatly augmented, consistent with SGC activation. TNF-α RNA level in DRG L6-L2 was elevated by 50%, and protein level increased by 84%. Similar changes in cytokines were observed previously in DRG (Huang et al., 2013) and TG, (Takeda et al., 2009). Song et al. claimed that the TNF was located in SGCs, but this requires further verification. Electrical stimulation of the axons of sensory neurons caused an increase of over 3-fold in the level of TNF-α in the bathing medium, indicating the release of this cytokine from the ganglion (presumably from SGCs). However, as TNF-α was believed to be present in the SGCs, it is not clear how neuronal firing led to the release of this cytokine, although it is possible to conceive a chain of events that can mediate this effect. Obviously, much remains to be done on this topic, and the main importance of this and related studies is to stimulate more interest in the potential role of neuron-SGCs interactions in GI pain. In another study on rats, Kannampalli et al. (2014) used TNBS to induce colonic inflammation and measured pain reactions electrophysiologically in response to colo-rectal distension. They found that following TNBS application both SGCs in DRG and microglia in the spinal cord were activated. Drugs known to inhibit glial activation (minocycline for microglia, and arabinofuranosyl cytidine for both microglia and SGCs), reduced pain reactions. This work again supports the idea that glial activation is a crucial element in the generation and maintenance of visceral pain, and that these cells can be an effective therapeutic target. Overall, the results from work on rats is in agreement with those on mice, supporting the general conclusion that SGCs in DRG are activated by colonic inflammation and might contribute to GI pain. Moreover, it appears that the changes that SGCs undergo in the GI pain models are quite similar to those observed in somatic and orofacial pain models. This suggests that despite the considerable diversity of pain syndromes and manifold pathways and mediators, the changes in sensory ganglia may be rather invariable, offering a generalized approach to therapy. Furthermore, it could be speculated that the proposed role of SGCs in GI pain, can be generalized to other types of visceral pain, but to establish this, further experimental work is required.

## The Role of Calcium Waves in Glia-Neuron Interactions

**Figure 6:**
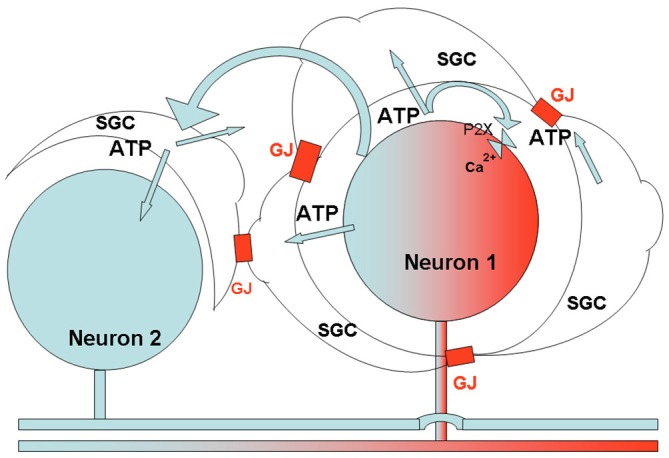
**Schematic diagram describing the proposed relations between SGCs and neurons in sensory ganglia, and the consequences of peripheral insults.** The axon of neuron one had been exposed to local injury, which causes various changes in the neuronal soma. One such change is increased firing of action potentials, which induces ATP release from the neuron and activates P2 receptors on SGCs surrounding the neuron, and on the neuron itself, causing an increase in [Ca^2+^]_in_ in these cells. Because of the enhanced sensitivity of SGCs to ATP (Blum et al., 2014; and Feldman-Goriachnik et al., 2015), this action will be augmented, and the higher level of [Ca^2+^]_in_ will lead to a greater release of ATP from the cells (Suadicani et al., 2010). ATP released from the SGCs and neuron, in combination with the augmented communication by gap junctions (GJ) following inflammation, will cause an increased spread of Ca^2+^ waves to a neighboring neuron (Neuron 2, which was not affected directly by the injury), and its surrounding SGCs (see Suadicani et al., 2010). The Ca^2+^ waves activate these cells, and the overall effect will be a greater neuronal excitation. This model can explain how peripheral injury can increase excitability of a large number of sensory neurons, which may contribute to chronic pain. The model is consistent with observations on the effects of insults in somatic, orofacial and visceral tissues.

We still know little on how SGC activation contributes to neuronal hyperexcitability, but it seems that the key to this question lies in a better understanding of the bidirectional neuron-SGC interactions. As little work has been done on this topic in relation to GI pain, most on the information must be derived from work on somatic and orofacial pain. A major mode of intercellular communication in sensory ganglia is chemical, and it was suggested that enhanced release of mediators (ATP, cytokines, glutamate, and others) after injury is an important factor in nociception (Takeda et al., 2009; Huang et al., 2013). A major mechanism of signal propagation within the glial network is intercellular calcium waves (ICW; Scemes and Giaume, 2006). ICW have been studied intensively in the CNS and it is established that they are mediated by both GJ and chemical mediators, most notably ATP (Scemes and Giaume, 2006). Channels, like pannexin1 (Iglesias et al., 2009), connexin hemichannels (Orellana and Stehberg, 2014), and P2X7 purinergic receptors (Baroja-Mazo et al., 2013), which allow the release of ATP and other mediators are likely to play a role as well. The only available information on ICW in sensory ganglia is from a study on TG cultures containing SGC and neurons (Suadicani et al., 2010), which showed that both GJ and the release of ATP from both neurons and SGCs are involved. We have suggested a model that incorporates these ideas to explain how enhanced ICW contribute to neuronal hyperexcitability in pain models (Kushnir et al., 2011; Hanani, 2012), see Figure 6.

## Nodose Ganglia and Pain

Vagal afferents innervate internal organs such as the heart, respiratory tract, and most of the GI tract (Browning and Mendelowitz, 2003; Undem and Weinreich, 2005; Li, 2007). The cell bodies of the sensory neurons that give rise to these afferents are located in the nodose and jugular ganglia. In mice and rats these two ganglia are fused and referred to as NG. A well known function of vagal afferents is to mediate reflexes such as swallowing, vomiting, and coughing (Undem and Weinreich, 2005). Another function of the vagi is the inflammatory reflex by which central vagal circuits are activated, leading to an attenuation of inflammatory processes (Pavlov and Tracey, 2012). The vagi appear to contribute to pain sensation in a complex manner (Jänig, 2005). On one hand, vagal activation leads to visceral pain, e.g., angina pectoris (Rosen, 2012) and pancreatic pain (Schwartz et al., 2013). Also, vagal afferent are important for pain associated with chemoreception, like sensing acidic pH and the presence of bile acids in the upper GI tract (Bielefeldt and Gebhart, 2013; Holzer, 2015). The electrical activity of vagal afferents was found to increase following injury to the stomach (Miranda et al., 2009), which might be associated with vagal-mediated pain. On the other hand, vagal activation can reduce somatic pain via central pathways (Miao et al., 2000; Jänig, 2005), and this is correlated with inhibitory actions of vagal afferents on spinothalamic neurons (Chandler et al., 1991).

## SGCs in Nodose Ganglia

The effects of GI tract inflammation on NG neurons has been studied by several groups (for example, Bielefeldt et al., 2002a,b; De Jonge et al., 2003; Dang et al., 2005; Banerjee et al., 2007; Hu et al., 2014). Overall, it appears that GI inflammation increases the excitability of NG neurons. Neurons in the NG, like in other sensory ganglia, are surrounded by SGCs. The few studies on this topic show that SGCs in NG are quite similar to those in other sensory ganglia. There is evidence for orexin receptors in SGCs in rat and human NG (Burdyga et al., 2003) and for BDNF receptors on SGCs in NG of rats (Wetmore and Olson, 1995). A calcium imaging study has shown that SGCs in rat NG are sensitive to glutamate and to ATP (via P2YR) and can release GABA (Shoji et al., 2010; Yokoyama et al., 2015). These authors claimed that GABA release from SGCs onto nodose neurons can inhibit neuronal activity. However, there is no evidence for GABA synthesis in SGCs in TG (see Vit et al., 2009), although infection of rat SGCs with GAD65, an enzyme that synthesizes GABA, led to inhibitory actions mediated by GABA release from SGCs on neurons (Vit et al., 2009). A calcium imaging study on cultured rat NG cells has shown that glial cells (presumed to be SGCs) respond to cholecystokinin (CCK, 1 μM), ghrelin, and leptin 100 nM (Avau et al., 2013). In our preliminary study on mouse SGCs in intact ganglia, where the identity of the cells is not in doubt, SGCs did not respond to leptin (10 nM) or to CCK (1 μM).

The only available study on changes in nodose SGCs in a pain model is by Feldman-Goriachnik et al. (2015), who induced systemic inflammation in mice by a single i.p. injection of the endotoxin lipopolysaccharide (LPS). This treatment induces pain and also causes profound changes in SGCs in DRG (Blum et al., 2014). The changes induced in nodose SGCs were very similar to those observed in DRG after the same treatment, which included augmented dye coupling between SGCs surrounding different neurons and upregulation of GFAP in SGCs. Also, calcium imaging showed that LPS caused increased sensitivity of SGCs to ATP, largely by upregulating responses via P2X receptors (mostly P2X2 and P2X7), as found for SGCs in TG following local inflammation (Kushnir et al., 2011). The expression of pannexin1 in SGCs in rat NG neurons has been described by Retamal et al. (2014), and we found that LPS induced a 2-fold increase in pannexin1 expression in SGCs in NG of mice (Feldman-Goriachnik et al., 2015), These changes persisted for up to 14 d after LPS injection. The possible correlation between these changes and the behavior of NG neurons has not been determined yet, but by analogy with studies on other sensory ganglia, it can be expected that the changes associated with SGC activation will augment neuronal excitability. It may be proposed that the model described above (Figure 6), which is based on augmented calcium waves, leading to greater neuronal excitation in pain models, also applies to NG, but experimental work to test this idea is required.

Altering vagal function has some important implications. In recent years vagus nerve stimulation (VNS) has been used to treat various serious neurological diseases (Hays et al., 2013) and also somatic (Kirchner et al., 2006) and GI pain (Chen et al., 2008) and migraine (Goadsby et al., 2014). If the electrical activity of vagal afferents can be controlled by altering SGC-neuron interactions using drugs, this could replace VNS, which requires the implanting of electrodes. Clearly, further research on SGCs in the NG in rodents and also in higher species is of great interest.

## Satellite Glial Cells in Sympathetic Ganglia

The sympathetic nervous system has been implicated in a variety of pain syndromes, and in particular complex regional pain syndrome (CRPS; Jänig, 2010; Schlereth et al., 2014). Peripheral injury induces the sprouting of sympathetic fibers in DRG (McLachlan et al., 1993), which may act as a link between the pain and sympathetic pathways. SGCs may be involved in this pathway, as it was found that in a colonic inflammation model, numerous SGCs contained the enzyme tryosine hydroxylase, which synthesizes norepinephrine (Xia et al., 2011). Neurons in paravertebral and prevertebral sympathetic ganglia are surrounded by SGCs that are rather similar to those in DRG (Hanani, 2010). The GI tract is supplied by sympathetic fibers from prevertebral ganglia, but very little research has been devoted to changes in SGCs in any sympathetic ganglia. We have shown that 10 d after colonic application of DNBS in mice, coupling among SGCs surrounding different neurons in prevertebral ganglia (superior mesenteric ganglia) increased by over 14-fold (Hanani et al., 2010). This adds to the evidence that SGCs in sympathetic ganglia can be activated by injury (Hanani, 2010). In analogy with the contribution of SGC activation to excitation of DRG neurons, it can be hypothesized that activation of SGCs in sympathetic ganglia contributes to excitation of sympathetic neurons, thereby augmenting sympathetic outflow into the pain pathways.

## Conclusions

It is now established that SGCs in sensory ganglia, like glial cells in the spinal cord, undergo activation in pain models and are an essential element within the pain pathways. Most of the available information on SGCs derives from work on somatic and orofacial pain models, but several recent studies have shown that in models of colonic inflammation SGCs in DRG are activated as well. This activation, and the functional changes that occur in SGCs apparently participate in the processes that augment neuronal activity, and therefore these cells are likely to contribute to GI pain. It will be interesting to explore this idea in animal models of inflammation in other parts of the GI tract. As cells in DRG are exposed to the circulation, SGCs could be an effective target for the treatment GI pain.

## Conflict of Interest Statement

The author declares that the research was conducted in the absence of any commercial or financial relationships that could be construed as a potential conflict of interest.
